# Balanced and unbalanced solutions modulate the release of Matrix Metalloproteinase-9 (MMP-9) from neutrophils in response to inflammatory stimuli: an in vitro study

**DOI:** 10.1007/s00011-014-0709-5

**Published:** 2014-01-24

**Authors:** Alessandro Trentini, Tiziana Bellini, Maria C. Manfrinato, Franco Dallocchio, Enrico Fainardi, Raffele Alvisi, Valentina Alvisi, Carlo A. Volta

**Affiliations:** 1 Department of Biomedical and Specialty Surgery Sciences, University of Ferrara, Luigi Borsari 46, 44121 Ferrara, Italy; 2 Neuroradiology Unit, Department of Neurosciences and Rehabilitation, Azienda Ospedaliera-Universitaria, Arcispedale S. Anna, Ferrara, Italy; 3 Department of Morphology, Surgery and Experimental Medicine, University of Ferrara, Ferrara, Italy

**Keywords:** Matrix Metalloproteinase-9, Neutrophil, Balanced solution, Unbalanced solution, Volume replacement solutions

## Abstract

**Objectives and design:**

We investigated the effect of balanced (BS) and unbalanced (UBS) solutions in the absence or presence of hydroxyethyl starch (HES) on neutrophil functionality, evaluating the release of matrix metalloproteinase (MMP)-9, myeloperoxidase (MPO), and MMP-8.

**Materials and methods:**

Neutrophils were isolated by gradient centrifugation and dextran sedimentation and incubated in BS or UBS without or with HES, in the absence or presence of Interleukin-8 (IL-8) or Lipopolysaccharide (LPS). MMP-9, MPO, and MMP-8 were assayed by commercially available ELISA kits.

**Results:**

There was not any influence of volume replacement solutions on the release of the enzymes from resting neutrophils. After IL-8 stimulation, the release of MMP-9 was higher in BS than in UBS or RPMI-1640, whereas HES enhanced its release regardless of the composition. After LPS stimulation, the release of MMP-9 was higher in both UBS and BS than RPMI-1640, but HES brought its release back to physiological conditions. No difference was found in the release of MPO and MMP-8 after stimulation with IL-8 or LPS.

**Conclusion:**

Volume replacement solutions might have an impact on the release of MMP-9 depending on the inflammatory milieu, suggesting that the use of balanced or unbalanced solutions is not a neutral choice.

**Electronic supplementary material:**

The online version of this article (doi:10.1007/s00011-014-0709-5) contains supplementary material, which is available to authorized users.

## Introduction

Fluid resuscitation is one of the most important aspects in the management of critically ill patients. Although evidence exists on the influence of different volume replacement regimens on systemic inflammation [1, 2], there are still controversial data regarding their effect on neutrophil functions [3, 4]. Moreover, nothing is known about the influence of balanced and unbalanced regimens on the release of Matrix Metalloproteinase-9 (MMP-9), a protease stored in tertiary granules of neutrophils and associated with post-operative complications [5] and worse outcomes during septic shock [6], and on the release of Myeloperoxidase (MPO) and Matrix Metalloproteinase-8 (MMP-8), stored in the primary and secondary granules of neutrophils, respectively. Therefore, we aimed to investigate the effect of balanced (BS) and unbalanced (UBS) solutions in the absence or presence of hydroxyethyl starch (HES) on the degranulation of neutrophils, by mimicking inflammatory conditions through interleukin(IL)-8 and lipopolysaccharides (LPS) treatments.

## Methods

Neutrophils were isolated from leucocyte-enriched buffy coats by Ficoll-Paque Plus gradient centrifugation (GE Healthcare, Milan, Italy) and dextran sedimentation, as described elsewhere [7]. Contaminating erythrocytes were removed by hypotonic lysis. The final cell pellet was suspended in RPMI-1640 medium (Sigma-Aldrich, Milan, Italy) supplemented with 50 U/ml penicillin, 50 μg/ml streptomycin.

Neutrophils (4 × 10^6^ cells) were suspended in 1 ml of either balanced (Sterofundin ISO, BBraun, Melsungen, Germany), or unbalanced (Sodium Chloride 0.9 %, Fresenius Kabi Italia, Italy) solution, HES (final concentration: 1.5 %) dissolved in balanced (Tetraspan, BBraun, Melsungen, Germany) or unbalanced (Amidolite, BBraun, Melsungen, Germany) electrolyte solution, or RPMI-1640 as a control. The cells were incubated for 30 min at 37 °C in slow agitation in the absence or in the presence of 10 μg/ml LPS (Sigma-Aldrich, Milan, Italy; this high concentration is required for the priming of neutrophils in the absence of serum) [8] or 20 ng/ml IL-8 (Millipore, Italy) and then centrifuged for 10 min at 1300 rpm and 4 °C. The supernatants were collected and frozen at −80 °C until their use.

The amounts of MMP-9, MPO, and MMP-8 released by neutrophils were measured using commercially available enzyme-linked immunosorbent assays (ELISAs) (Amersham, human MMP-9, Biotrak ELISA system; Innozyme Myeloperoxidase Activity Kit, Calbiochem, cat. CBA024; MMP-8 Human ELISA kit, Abcam, cat. Ab100609) according to the manufacturer instructions. The results are presented as mean ± standard deviation of five experiments. Statistical differences among experimental groups were calculated using a Student’s *t* test with the Bonferroni correction for multiple comparisons; *P* values < 0.05 were classified as statistically significant.

## Results

Neutrophils were suspended either in balanced or unbalanced solutions in the absence or presence of 1.5 % HES (final concentration) or in RPMI-1640 as a control, either without any stimuli or stimulated with IL-8 or LPS, and the levels of MMP-9, MPO, and MMP-8 were evaluated.

### Levels of enzymes released from resting neutrophils

**Fig. 1:**
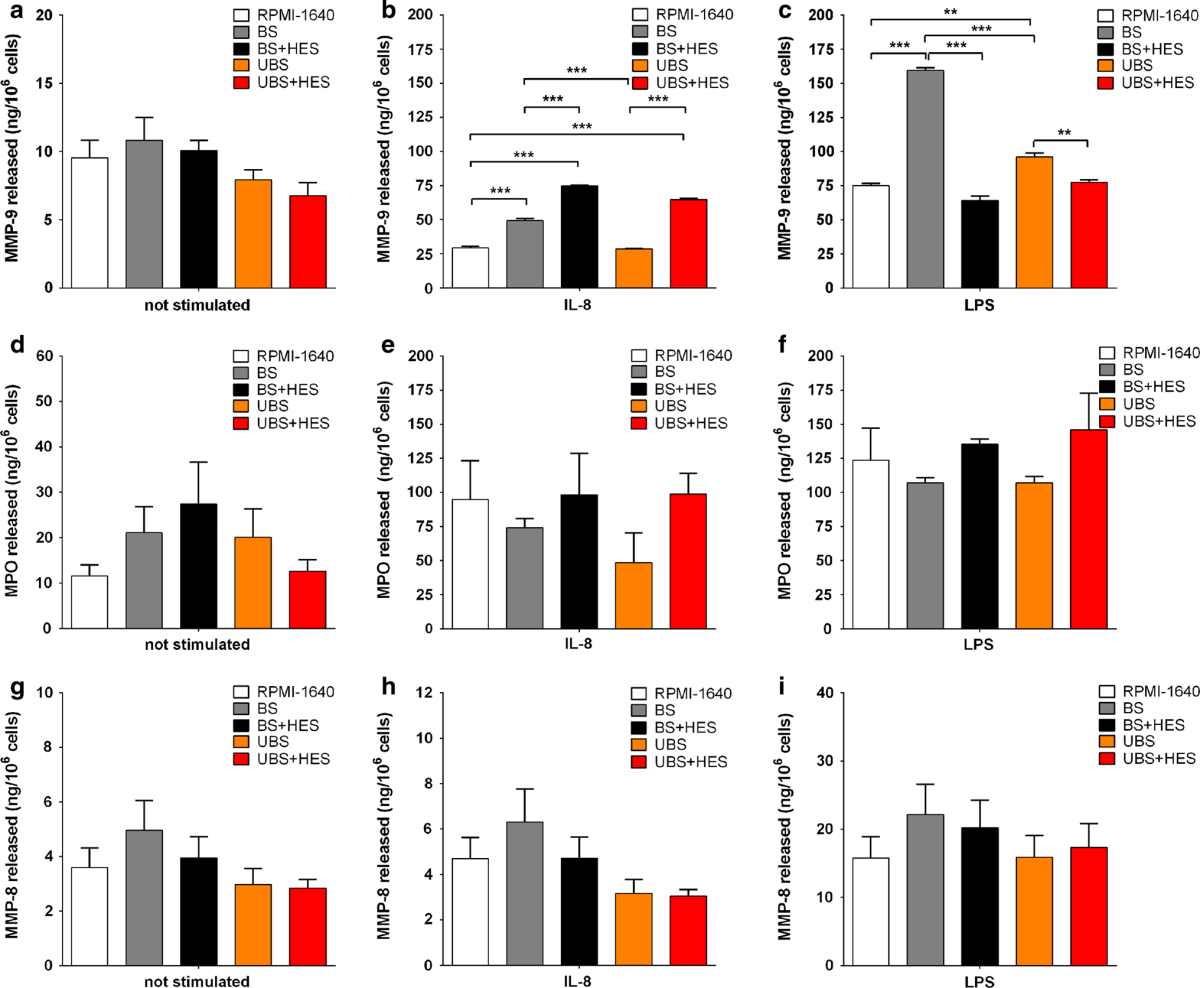
Levels of MMP-9 (*panels* **a**–**c**), MPO (*panels* **d**–**f**), and MMP-8 (*panels* **g**–**i**) released from neutrophils not stimulated or stimulated with IL-8 or LPS. Neutrophils were placed in balanced or unbalanced solutions in the absence or in the presence of 1.5 % HES or in RPMI-1640 as a control, not stimulated (*panels* **a**, **d** and **g**) or stimulated with IL-8 (*panels* **b**, **e** and **f**) or LPS (*panels* **c**, **f** and **i**) as described in the “Methods” section. The values in the graphs represent the mean ± SD. ** = *P* < 0.01; *** = *P* < 0.001. *BS* balanced solution, *UBS* unbalanced solution, *BS* *+* *HES* hydroxyethyl starch dissolved in balanced solution, *UBS* *+* *HES* hydroxyethyl starch dissolved in unbalanced solution

As shown in Fig. 1 (panels a, d and g), we did not find any difference in the release of MMP-9, MPO, or MMP-8 from neutrophils placed in the different solutions in the absence of stimuli, although a trend (not significant) of higher levels of MMP-9 and MMP-8 was observed when neutrophils were suspended in BS compared to UBS.

### Stimulation with IL-8

As shown in Fig. 1b, there was a general increase in the release of MMP-9 in all the different volume replacement solutions compared to the RPMI-1640 control (considered as the physiological situation), except when neutrophils were placed in UBS, which showed levels of MMP-9 comparable to the control.

Interestingly, when neutrophils were placed in either BS or UBS combined with HES, there was a large increase in the release of MMP-9 with respect to their saline counterpart (Fig. 1b, *P* < 0.001 for both regimens), suggesting that HES potentiates the response of neutrophils during IL-8 stimulation. Moreover, we observed a higher release of MMP-9 in the presence of a balanced regimen compared to an unbalanced one (Fig. 1b, *P* < 0.001, grey vs. orange bar; *P* < 0.01, black vs. red bar).

On the contrary, we did not find any difference in the release of MPO or MMP-8 in any of the volume replacement solutions when neutrophils were stimulated with IL-8 (Fig. 1 panels e and h).

### Stimulation with LPS

The release of MMP-9 after LPS stimulation is reported in Fig. 1c. As evidenced, there was an increase in the release of MMP-9 in both BS and UBS compared to the control, though it was more marked in BS (Fig. 1c, *P* < 0.001, grey vs. open bar). Thus, the release of the enzyme was dependent on the composition of the solution since the BS showed higher levels of MMP-9 than the UBS (159.5 ± 1.9 ng/10^6^ cells and 96.0 ± 2.8 ng/10^6^ cells, respectively).

On the other hand, HES brought the release of MMP-9 back to the physiological situation (Fig. 1c, black and red bars vs. open bar), as evidenced by the lack of significance between these treatments. Instead, we did not find any difference in the release of MPO or of MMP-8 from neutrophils suspended in the different volume replacement solutions (Fig. 1 panels f and i).

## Discussion

In the present study, we evaluated the effect of BS and UBS in the absence or presence of 1.5 % HES, the average concentration reached during the treatment of hypovolemia corresponding to 1 L of 6 % HES administered in a patient of 70 Kg, on the release of MMP-9, MPO, and MMP-8 from neutrophils. At this scope, we used two stimuli that might reflect different inflammatory conditions: IL-8, which increases during the postoperative period [9], and LPS, which mimics the presence of bacteria.

The main results of our in vitro study show that the composition of the solution (balanced or unbalanced) as well as HES can modulate the release of MMP-9 from neutrophils depending on the used stimulus.

In particular, we observed that the minimum release of MMP-9 occurred in the unbalanced solution after IL-8 stimulation and in balanced or unbalanced solution added with HES in the presence of LPS. Moreover, we did not find any difference in the release of the enzyme in the absence of stimuli, suggesting that infusional solutions might be able to influence the neutrophil’s response only if subjected to stimulation. The effect of the ionic composition might be explained considering that the variation of ion concentrations can impair neutrophil’s degranulation [10–12] as well other functions [13]. Of note, we did not observe any influence of the volume replacement solutions on the release of MPO nor MMP-8, contained in the primary and secondary granules of neutrophils, respectively, from resting and stimulated neutrophils, therefore suggesting a specific influence on the release of tertiary granules.

Translating our in vitro results to clinical practice, it might be useful to administrate a UBS during the perioperative period in order to minimize the activation of polymorphonuclear leukocytes. On the other hand, it is known that unbalanced solutions show several side effects [14]. Thus, other aspects besides the activation of neutrophils must be considered in the choice of the solution to administer.

On the contrary, in the presence of LPS, which might mimic a septic shock, a balanced solution with added HES should be administered in order to avoid an over activation of polymorphonuclear cells. In fact, this might lead to a massive release of MMP-9, which in turn may contribute to the vascular leakage observed during sepsis [6]. Indeed, human neutrophils release TIMP-free MMP-9 [15], which might be massively activated by other proteases present in the extracellular space as well as by cathepsins released by neutrophils, acting as an uncontrolled effector of tissue damage [16]. Therefore, the observed modulation could be of large impact in the treatment of patients requiring a large fluid administration, or in situations where a systemic inflammatory response is triggered. The lack of in vivo data supporting these hypotheses might be a limitation of this study. However, we recently found that volume replacement solutions are able to modulate the systemic inflammatory response by tuning the levels of the anti-inflammatory cytokine IL-10 and by acting on the activation cascade of MMP-9 as well as its inhibitor levels [2].

In conclusion, different volume replacement solutions might have divergent impacts on the release of MMP-9 from neutrophils, depending on the inflammatory milieu. However, further basic research is needed to elucidate and understand the mechanisms underlying the effect of both ionic composition and HES on neutrophil degranulation.

## Electronic supplementary material

Below is the link to the electronic supplementary material.

Supplementary material 1 (DOCX 12 kb)
