# Supplementary material for: Balanced and unbalanced solutions modulate the release of Matrix Metalloproteinase-9 (MMP-9) from neutrophils in response to inflammatory stimuli: an in vitro study
Source: Inflamm Res. 2014 Jan 24;63(5):325–8. doi: 10.1007/s00011-014-0709-5 (PMC3983875; doi:10.1007/s00011-014-0709-5)
Supplement: Supplementary file 1 — Supplementary material 1 (DOCX 12 kb) [file 11_2014_709_MOESM1_ESM.docx]

**Supplementary Methods**

***Determination of MPO***

The concentration of MPO released by neutrophils was measured using a commercially available ELISA (Innozyme Myeloperoxidase Activity Kit, Calbiochem, cat. CBA024) according to the manufacturer instructions. All reagents and standards were included in the kits.

Briefly, 100 µl of each sample diluted 1:10 were pipetted in duplicate into 96 microwells microtiter plate precoated with anti-MPO antibody. Seven dilutions of standard were dispensed in duplicate in a range of 5 - 100 ng/ml and dilution buffer into two wells as negative control (blank). After 1 hour of incubation at RT and 4 washing cycles, 100 µl of TMB working solution were added to each well and incubated at 37 °C for 30 minutes. At the end of the incubation, the reaction was stopped by the addition of 100 µl of stop solution and the absorbance read at 450 nm in a microtitre plate reader spectrophotometer (Infinite M200, Tecan, Italy).

***Determination of MMP-8***

The concentration of MMP-8 released by neutrophils was measured using a commercially available ELISA (MMP8 Human ELISA kit, Abcam, cat. Ab100609) according to the manufacturer instructions. All reagents and standards were included in the kits.

Briefly, 100 µl of each sample diluted 1:50 were pipetted in duplicate into 96 microwells microtiter plate precoated with anti-MMP-8 antibody. Seven dilutions of standard were dispensed in duplicate in a range of 8.23 - 6000 pg/ml and dilution buffer into two wells as negative control (blank). After 2.5 hours of incubation at RT and 4 washing cycles, 100 µl of biotinylated antibody were added to each well and incubated at RT for 1 hour. At the end of the incubation and after 4 washing cycles, 100 µl of Streptavidin-HRP were added to each well and incubated at RT for 45 minutes. Afterwards, the plate was washed 4 times with wash buffer and 100 µl of TMB working solution were added to each well and the plate incubated at RT for 30 minutes in the dark. At the end of the incubation, the reaction was stopped by the addition of 50 µl of stop solution and the absorbance read at 450 nm in a microtitre plate reader spectrophotometer (Infinite M200, Tecan, Italy).
